# Thermal Compensation of Low-Cost MEMS Accelerometers for Tilt Measurements

**DOI:** 10.3390/s18082536

**Published:** 2018-08-02

**Authors:** Giuseppe Ruzza, Luigi Guerriero, Paola Revellino, Francesco M. Guadagno

**Affiliations:** Department of Science and Technology, University of Sannio, 82100 Benevento, Italy; giuseppe.ruzza@unisannio.it (G.R.); paola.revellino@unisannio.it (P.R.); guadagno@unisannio.it (F.M.G.)

**Keywords:** MEMS, accelerometer, compensation, polynomial equation, thermal chamber, monitoring, tilt measurement, Arduino^®^

## Abstract

Low-cost MEMS accelerometers have the potential to be used in a number of tilt-based monitoring applications but have the disadvantage of being very sensitive to temperature variation (thermal drift). In this paper, we analyze the thermal behavior of a low-cost sensor in the range −10 to +45 °C in order to provide a simple compensation strategy to mitigate this problem. For sensor analysis, we have developed a miniaturized thermal chamber, which was mounted on a tilting device to account for tilt angle variation. The obtained raw data were used to construct low degree polynomial equations that by relating the measurement error induced by thermal drift (i.e., acceleration residuals) to temperature and inclination (of each specific axis), can be used for thermal compensation. To validate our compensation strategy, we performed a field monitoring test and evaluated the compensation performance by calculating RMS errors before and after correction. After compensation, the RMS errors calculated for both the X and Y axes decreased by 96%, indicating the potential of using a simple set of equations to solve common drawbacks that currently make low-cost MEMS sensors unsuitable for tilt-based monitoring applications.

## 1. Introduction

MEMS accelerometers have a wide range of applications in consumer electronics and are of common use in environmental and geotechnical applications. Environmental applications include sea wave monitoring and trees movement analysis. Geotechnical applications include structural health monitoring (e.g., bridges, railways, buildings, etc.) by fundamental frequency analysis, tunnel deformation and lining control, ground deformation monitoring, landslide displacement monitoring and early warning etc. For instance, the authors of [1] used a MEMS accelerometer to derive tree properties like mass variation and interception to precipitation by monitoring tree sway movement in response to external forcing. The authors of [2] used a MEMS-based monitoring system to analyze railway response to passing trains and assess changes in track health. The authors of [3] derived ground subsidence caused by the construction of the South Hongmei Road super highway tunnel in Shanghai from MEMS-based ground tilting measurements. The authors of [4] calculated the global deformation of a tunnel segment measuring tilt angles at particular locations along the segment with micro accelerometers. The authors of [5] used MEMS-based tilt-sensors, associated with volumetric water content sensors, to develop a landslide early warning system.

High precision monitoring application are often based on traditional electromechanical sensor since they have high accuracy and measuring resolution, which is nearly linear and constant through the measuring range, and high measuring stability [6,7]. Major disadvantages of these sensors are their high-power consumption, large size and their cost. For instance, a single electromechanical accelerometer, without the acquisition system, sells for a few to several thousand euros. Monitoring systems used for infrastructures, landslides and environmental control are often based on tilt measurements and need to be composed of a large number of sensors, which is a function of the dimension and the complexity of the problem. Thus, the final cost of the system could be very high. That’s why the number of installed sensors is often limited, or monitoring is not applied at all for apparently simple problems. In this context, recently developed low-cost MEMS accelerometers and other low-cost sensors provide an opportunity to overcome this drawback, also considering that they are particularly suitable for tilt measurement [8]. In fact, they are being increasingly used in monitoring applications also in association with open-source controlling platforms such as Arduino^®^ [9,10,11,12].

Despite advantages over traditional high precision electromechanical sensor such as smaller size and power consumption and sufficient resolution for many monitoring problems, MEMS accelerometers have the disadvantage to be very sensitive to temperature variations [13]. This sensitivity is connected to the sensors’ design and constitutive materials and is related to the physical deformation of the sensor [14,15,16], which causes a variation of the electronic capacitance that modifies the acceleration signal. If the temperature exceeds the limits suggested by manufacturers, the deformation would even lead to the destruction of the sensor. Such sensitivity can, thus, generate a linear/nonlinear thermal drift that induces a potential and variable error in the measurement of the acceleration that needs to be analyzed and corrected [17,18,19,20]. For high precision and expensive sensors, this effect is often mitigated through onboard hardware or software-based solutions. In the case of low cost sensors, thermal drifting is often not assessed and needs to be mitigate in pre- or post-processing. Additionally, these sensors, can suffer from full/partial measuring offset (also connected to the sensor cabling system) [21] that, for high accuracy application, needs to be corrected as well.

In this paper, we present a study of the thermal properties of a low-cost capacitive LSM9DS0 MEMS IMU (STMicroelectronics, Geneva, Switzerland) containing three accelerometric sensors and provide a compensation strategy to correct typical drifting caused by temperature variation. The idea behind the paper is to underline the increased potential of low cost sensors for multiple environmental monitoring applications providing simple tools for measurement improvement. In this perspective, the analyzed sensor is being used for the development of a tilt-based landslide monitoring/early warning system that will be presented in a forthcoming paper. For calibration purposes, we developed a dedicated miniaturized Arduino^®^-based thermal chamber that allow to simulate temperature variation in controlled conditions. Additionally, to understand if the thermal drifting is modulated by the magnitude of the inclination, the thermal chamber was mounted on a tilting device. We used calibration data to derive specific compensation polynomial equations (i.e., surfaces of compensation) for each axis of the accelerometer and estimate compensation performance through RMS error evaluation. This strategy can be applicable to all capacitive low-cost MEMS sensor in order to make them applicable to a number of tilt-based monitoring conditions where medium to high precision and stability is needed.

## 2. The Low-Cost MEMS IMU

**Figure 1:**
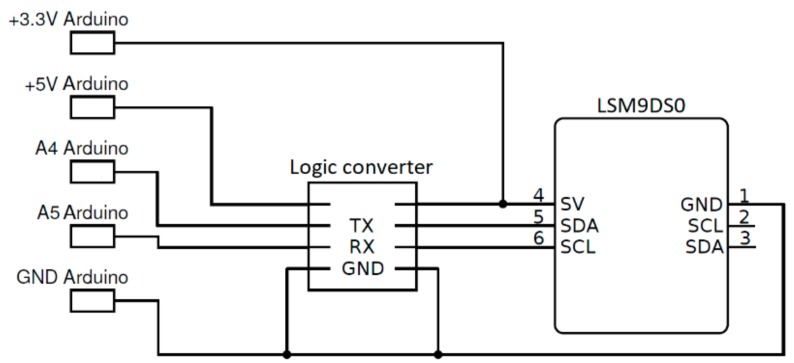
Wiring diagram of the LSM9DS0 IMU.

For our project, we use a LSM9DS0 MEMS IMU, also known as INemo^®^, that is formed of three magnetometers, three accelerometers, three gyroscopes and a temperature sensor. The IMU is equipped with an onboard 16-bit ADC so that onboard sensors can be read through the I2C or SPI interfaces. Onboard accelerometers can be set up to work in a range of ±2, ±4, ±6, ±8 or ±16 g [22]. The sampling frequency is customizable in a range between 3.125 and 1600 Hz. Analog supply voltage is between 2.4 V and 3.6 V and the power consumption is of about 7 mA. We choose this accelerometer because it has a resolution of 0.061 mg/ Least Significant Bit (LSB) in the measuring range of ±2 g and an operating range from −40 to +85 °C. This sensor was connected to an Arduino^®^ Nano board using the I2C communication interface [23] and the wiring diagram is reported in Figure 1. Due to a difference in operating voltage between the IMU and the Arduino^®^, a bi-directional logic converter was used.

**Figure 2:**
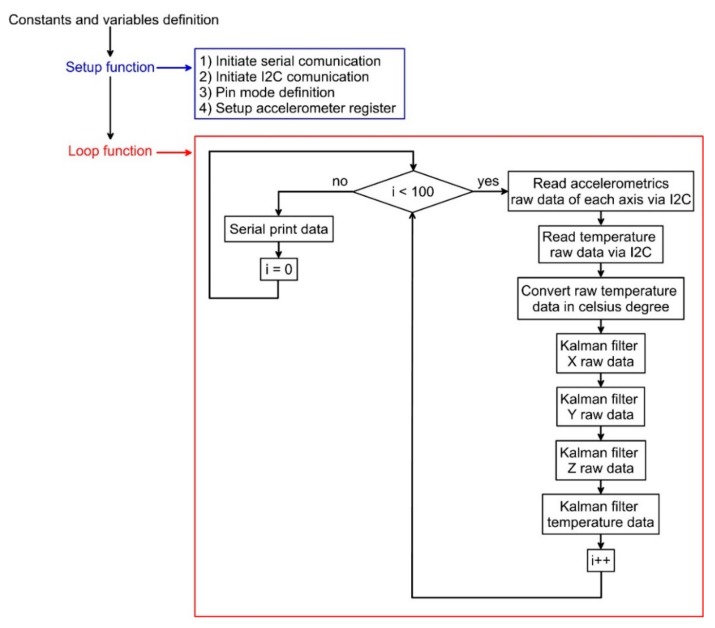
Flow chart showing the structure of the code used for reading the LSM9DS0 onboard accelerometers.

Figure 2 shows the logic of the code (reported in the Supplementary File A) used for reading and communicating with the onboard accelerometers of the LSM9DS0 IMU. Reading parameters are reported in Table 1. For our analysis, we used a sampling rate of 100 Hz and, since ADCs operate with a constant sampling frequency and considering the Nyquist theory about signals digitization [24,25], an antialiasing threshold of 50 Hz. After parameter setup, raw data of each accelerometer and temperature sensor were read through the I2C protocol (X, Y, Z and temperature), pre-processed using a discrete, single-stage Kalman filter and decimated to reduce the number of records [26,27,28,29,30,31]. 

Kalman parameterization was completed using a trial and error procedure. In particular we used a Q parameter of 1 × 10^−8^ and a R parameter of 0.01. Raw temperature data was converted into Celsius degrees considering a conversion factor of 8 LSB/°C and, to solve a problem of measurement offset, we introduced an additional corrective coefficient, estimated in 21.00 °C, that needs to be added to the converted value. This coefficient was estimated under laboratory-controlled conditions.

**Figure 3:**
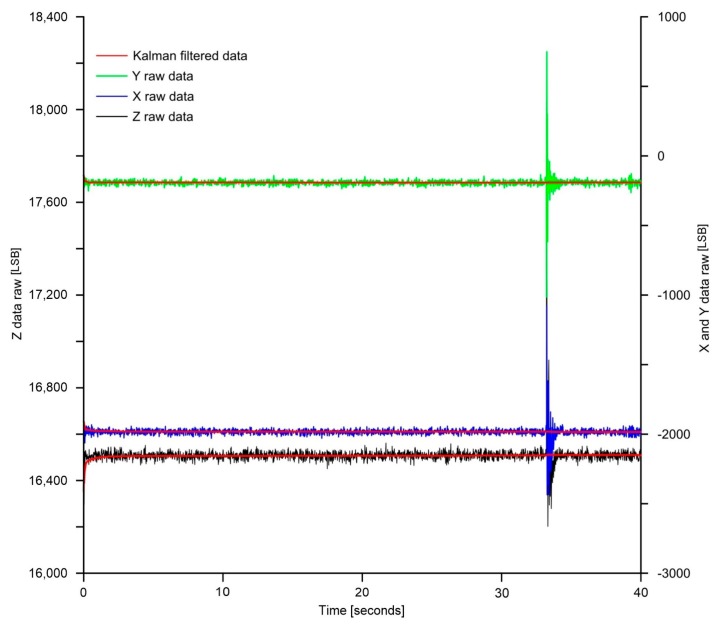
Comparison between raw data and filtered data.

Figure 3 shows the effect of the Kalman filter on raw data in the absence and presence of impulsive perturbation. From this figure, it is clear that the filter was able to remove both the white noise and the impulsive perturbation that was simulated after ~35 s from the beginning of the experiment. This was possible due to a correct setup of the characterizing parameters (R and Q).

## 3. Methods

### 3.1. The Miniaturized Thermal Chamber

**Figure 4:**
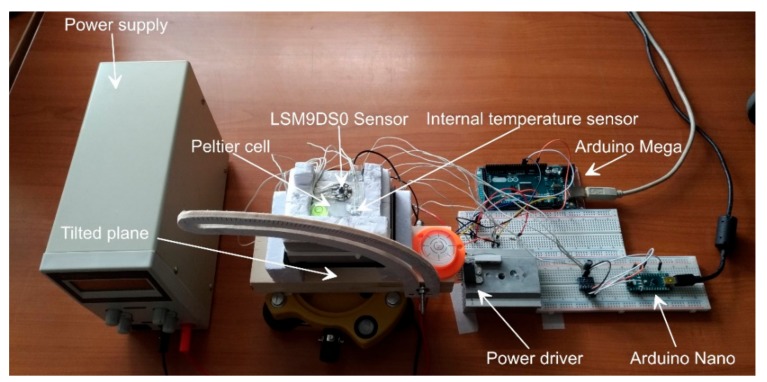
The thermal chamber.

**Figure 5:**
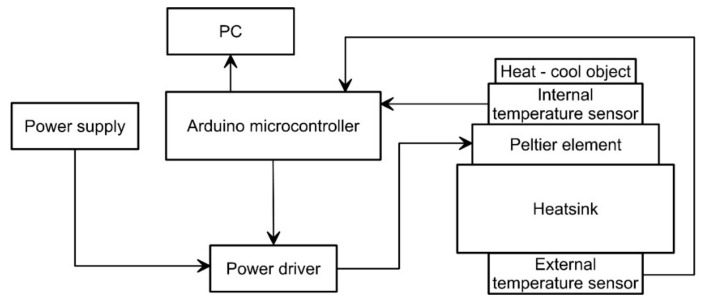
Block diagram of the designed equipment.

To simulate temperature variation, we have developed a low-cost miniaturized and tiltable thermal chamber. The chamber, shown in Figure 4, is composed of: (i) a thermoelectric cooling and heating element, (ii) a power driver, (iii) temperature sensors, and (iv) a control board. A detailed scheme of the chamber is shown in Figure 5.

The thermoelectric element is a Peltier cell [32]. For our development, we used a TEC1-12706 Peltier element (Hebei I.T., Shanghai, China) mounted on a heatsink to improve heat dissipation. It is characterized by a maximum power of 50 W, a maximum temperature difference between the faces of 66 °C, a maximum current of 6.4 A and a maximum voltage of 14.4 V. To modulate heat flux and consequently allow full range temperature variation on a single face of the cell, we used a power drive based on a dual full-bridge [33] L298N driver, which is an integrated 15-lead Multiwatt and Power SO20 monolithic package [34]. This device, using standard TTL logic, allows to control the Peltier cell through a microcontroller.

**Figure 6:**
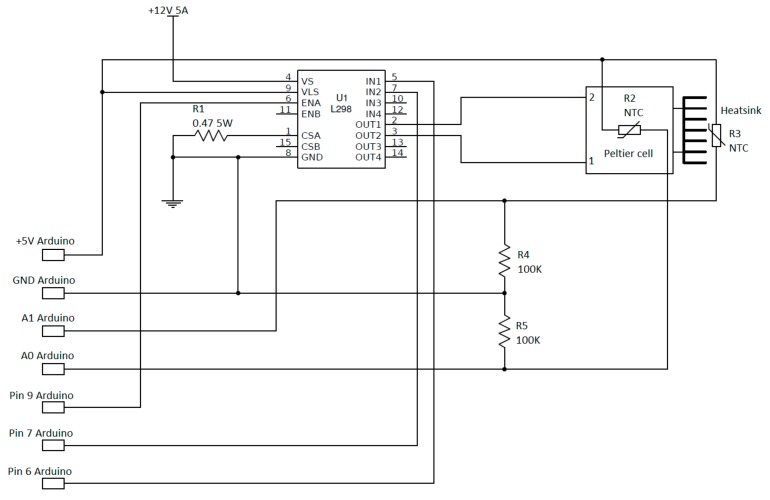
Wiring diagram of the thermal chamber.

Figure 6 shows the wiring diagram of the thermal chamber and its connection with the Arduino^®^ board, in this case, an Arduino^®^ Mega 2560 board (https://www.arduino.cc). Cell power is controlled using Pulse Width Modulation [35,36] technique (PWM), which allow to turn power on and off the device very quickly. Power modulation occurs through the ENA input pin of the L298N driver, while current flux direction is controlled by the IN1 and IN2 input pins as shown in Figure 6. These pins are connected to the Arduino^®^ digital outputs 9, 7, 6 pins. Temperature control within the chamber is completed using two CLW1064 100 KΩ thermistors (TDK EPCOS, Munich, Germany), characterized by a tolerance of ±1%, installed with a reference resistor of 100 KΩ (tolerance of 5%, thermal time response lower than 5 s). Each thermistor is installed on a face of the cell using a thermal compound. This is needed because the temperature of a side depends on the temperature of the opposite face, thus it is essential to control both sides of the cell. For temperature estimation we used the following equation [37]:

1/*T* = (*ln*(*R_t_*/*R*_0_)/*β*) + (1/*T*_0_) (1)

where *R*_0_ is the resistivity of the reference resistor (100 KΩ ± 1% at 25 °C) and *β* is the material constant provided by the manufacturer (*β* = 3950 K). The obtained temperature (*T*) is measured in Kelvin and needs to be converted in °C. Additionally, we used a polystyrene cover to homogenize and stabilize the temperature within the cell and minimize thermal dispersion.

**Figure 7:**
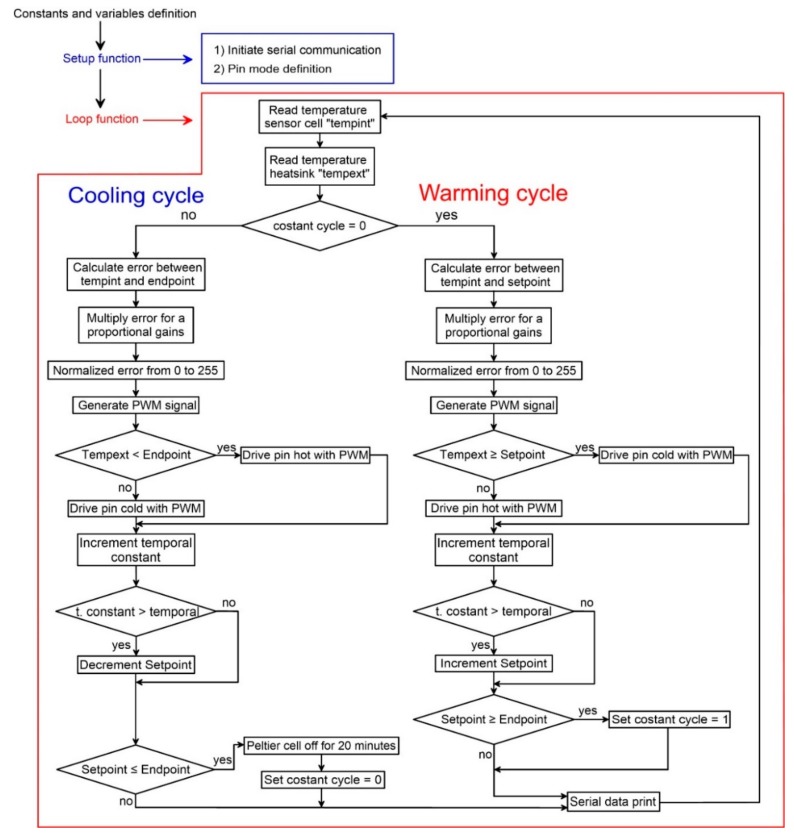
Flow chart showing the structure of the code used to control the thermal chamber.

The logic of the code (reported in the Supplementary File B) developed for making the chamber working is reported in Figure 7. The code, developed and compiled using the Arduino IDE environment, includes a two-way temperature cycle, a warming cycle and a subsequent cooling cycle. We arbitrary started from the warming cycle. The code is based on a simplification of the Proportional-Integral-Derivative algorithm (PID) [38] that uses only the *P_t_* of the PID algorithm [39] to reduce the steady state error of the system as a function of the gain factor:

*P_t_* = *K_p_e_t_* (2)

In this equation, *K_p_* is the proportional gain and *e_t_* is the error at time “*t*”. As reported in Figure 7, the first step after system setup (setup function) is the estimation of the internal and external temperatures as the average of two successive measurements. Subsequently, on the base of the estimated difference between the temperature of the reference face of the cell and the programmed temperature, the controller calculates the power needed to increase or maintain the programmed temperature. This value is calculated multiplying the temperature difference and the proportional gain, which is set at 350.

The proportional gain value was chosen using a trial and error calibration procedure and the final selected value corresponds to the best compromise between the velocity in reaching the programmed temperature and the difference between the programmed and simulated temperature for each single calibration step. Since we used an 8-bit PWM-controlled power drive, the temperature difference needs to be converted in a value between 0–255.

In other word, adopting higher values of the PWM the driver increases the relative duration of maximum power supply to the cell. After temperature stabilization for the predefined time interval the temperature of the reference face is shifted up modulating the power. When a single measuring cycle is ended, the controller checks if the programmed maximum temperature of the experiment is reached and, if so, it passes to the cooling cycle decreasing the temperature step by step. At the end of the cooling cycle the cell is stopped for 20 min before to start a new cycle.

**Figure 8:**
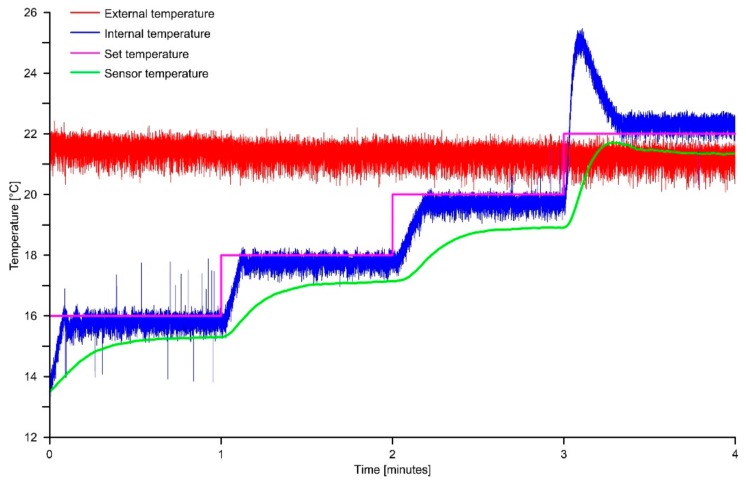
Example of temperature variation in the chamber during a warming experiment.

Figure 8 shows an example of temperature variation during a 4-step warming cycle. The duration of each measuring step is of 1 min and total temperature variation is of 6 °C (16 to 22 °C). In this example the programmed temperature increase is of 2 °C for each step. As shown in the graph of Figure 8, the temperature variation of the reference face of the Peltier cell (blue curve: Internal temperature), is consistent with the programmed variation. The temperature change between two consecutive warming steps of 2 °C is completed in approximately 13 s. It is important to notice that a temperature offset of about 0.5 °C has been observed between the programmed temperature and that measured on the reference face of the cell. This offset is negative for a temperature lower than the external and it is positive for higher temperatures. Figure 8 suggests also that when the internal temperature grow over the external, heat flux inversion induce an initial overheating of the reference face that is materialize by a transient overshoot. 

Additionally, it is possible to observe a consistent negative offset of the onboard temperature sensor of the LSM9DS0 IMU. Since our purpose is to use the temperature measured by the LSM9DS0 onboard sensor to compensate the accelerometric measurement, we use this value as it is.

### 3.2. Thermal Calibration Analysis

Multiple thermal simulations were completed to analyze the behavior of the onboard accelerometric sensors and obtain calibration data during positive and negative temperature variations. For our analysis, we chose a temperature range of −10 to 45 °C and simulate both warming and cooling cycles. This is in order to identify and compensate possible thermal hysteresis of the onboard sensor. The temperature range was chosen to be representative of field measuring condition in most of underground and subaerial applications. Each cycle consists of multiple steps characterized by a specific temperature (see purple line in the graph of Figure 8) with a variation between two consecutive steps setup at 2 °C. Each measuring step was completed in one minute. We used the titling device of the chamber to simulate sensor response at different inclination of each axis. In this way, we made multiple simulations in the inclination range from 5 to 45° with a 5° step. In total, we completed 37 simulations and the first was made with the sensor in the horizontal position (e.g., Xinc = 0°; Yinc = 0°; Zinc = 90°).

**Figure 9:**
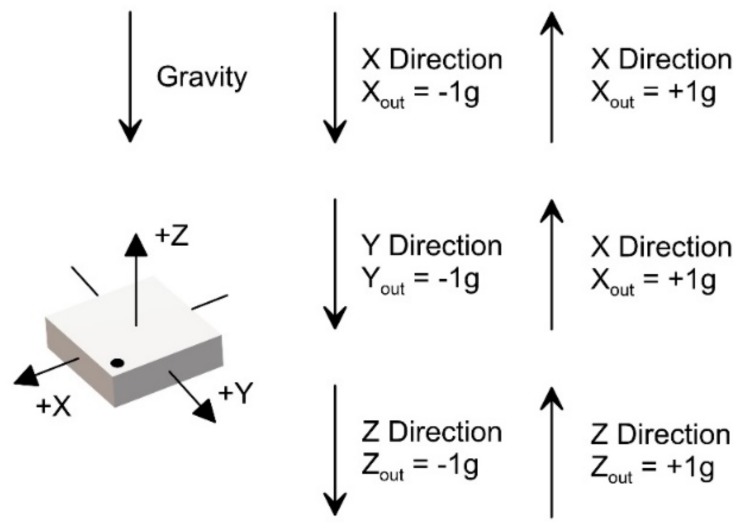
Standards for onboard accelerometric sensor reading and calculation.

The calibration procedure was completed registering the raw data of the accelerometers and the internal temperature sensors for a complete thermal cycle, for each inclination angle. This procedure was carried out by varying the X and Y angle alternately. Once the measurements for the positive directions of the +X and +Y angles were completed, those for the negative −X and −Y directions were made [40] (see Figure 9 for measurement standards). In this way, we obtained a dataset for the entire range −45 to +45° for both the X, Y and the Z axes.

### 3.3. Thermal Drifting Compensation and Testing

To compensate the onboard accelerometers’ drift as function of temperature variation and inclination, we fitted polynomial equations (i.e., surfaces) on the acceleration residuals (dependent variable), temperature and raw inclination (independent variables) data. Raw acceleration data, acquired during every single thermal simulations at specific inclination, were used to calculate residuals as difference between the measured acceleration at different temperatures and the reference acceleration at 25 °C. We chose this value as reference following the indication of the manufacturer. In this way, residual at 25 °C equals to zero. Acceleration residuals, temperature and inclination data were used to fit two 2nd-order polynomial equations (representing surfaces of compensation) for the X and Y axes, respectively. A first for data derived from the warming cycle and a second for data derived from the cooling cycle.

The Z axis was not considered in this analysis, since it is not as heavily affected by thermal drift as the X and Y axes. Additionally, due to its particular behavior, it is not always used in monitoring applications (i.e., two axis tilt measurements). Surface parameterization was automatically completed using cftool implemented in Matlab™ after choosing the type and degree of the fitting equation. Since open-source platforms like Arduino^®^, often used as basis of low cost monitoring systems, have a limited computational capacity, polynomial order was chosen to be as low as possible considering also the final RMS error. Best fit 2nd-order surface parameters were selected considering the lowest RMS error. It is important to notice that such surfaces were reconstructed with the aim of compensating only thermal drifting. Measurement offset was not considered in this analysis because monitoring application are often based on differential measurement of the reference parameter. Anyway, for monitoring applications that require the use of absolute measurement, our calibration provide the basis for offset correction.

Polynomial equations derived from this procedure have been used to test the compensation performance after the acquisition of raw data in natural environmental conditions. Especially, we made a first 3 h acquisition test (from 7 a.m. to 10 a.m. of 25 March 2018) with the sensor, housed within a plastic black box, in a fixed horizontal position. This allow the temperature to increase up to 48 °C simulating common warming field monitoring conditions. Subsequently, we made a second 9 h acquisition test (from 8 p.m. to 5 a.m. of 2 July 2018) to simulate common cooling field monitoring conditions with the sensor in fixed position (in this case the perfect horizontality was not guaranteed). In this second case, since measurements were made in July, the temperature variation was limited to approximately 10 °C (from 27 to 17 °C). Raw data were post-processed applying the polynomial equations in Matlab™ to evaluate compensation performance and RMS error variation in comparison with raw data. Additionally, to show the behavior of the system during alternation of warming and cooling thermal cycles and its stability despite the thermal hysteresis, we made a further 12 h test (from 8 a.m. to 8 p.m. of 9 July 2018). 

## 4. Results and Discussions

### 4.1. Thermal Behaviour of Onboard Accelerometer

**Figure 10:**
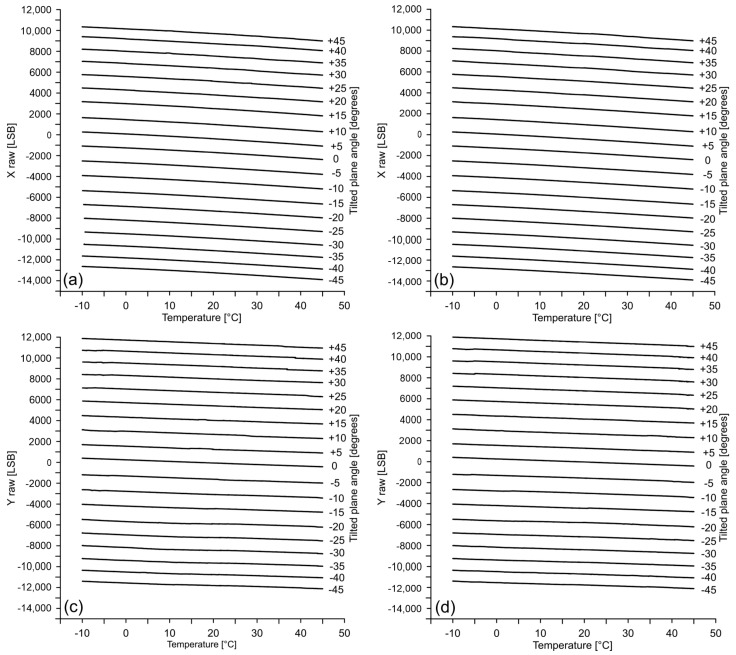
Graphs showing the raw acceleration measured along the (**a**,**b**) X and (**c**,**d**) Y axes at different inclinations during warming and cooling cycles, respectively.

**Figure 11:**
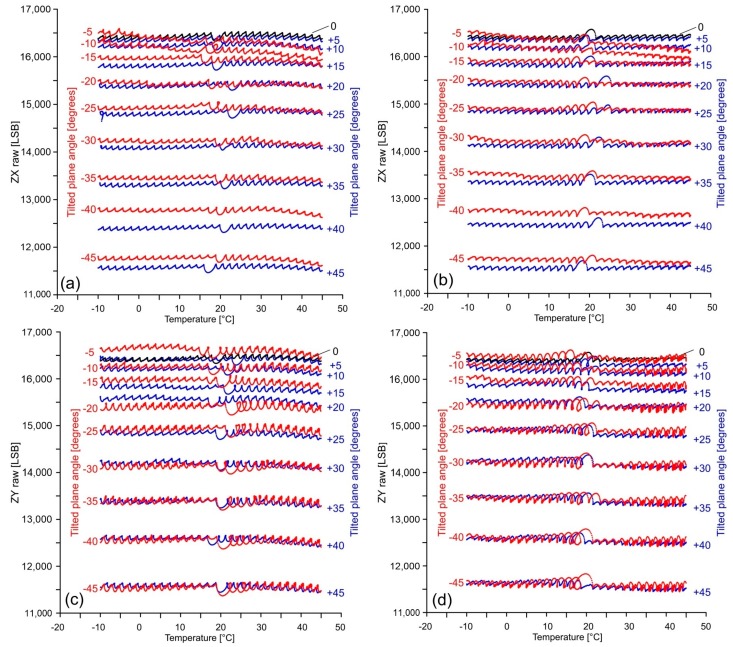
Graphs showing raw acceleration measured along the Z axis for different inclinations of the (**a**,**b**) X and (**c**,**d**) Y axes, during warming and cooling cycles, respectively.

Figure 10 and Figure 11 show raw acceleration data measured along the X, Y and Z axes during a complete thermal simulation (see the Methods section for parameterization). Graphs (a) and (b) of Figure 10 show raw data recorded along the X axis of the accelerometer during: (a) warming cycles and (b) cooling cycles. Similarly, graphs (c) and (d) show raw data recorded along the Y axis during warming and cooling cycles, respectively. Each line in the graphs represents the response of the axis for a different inclination. The graphs of Figure 11 show raw data recorded along the Z axis for different inclination of the X and Y axes. In particular, graphs (a) and (b) show the raw acceleration during warming and cooling cycles, respectively, at varying inclination of the X axis. Graphs (c) and (d) show the same data at varying the inclination of the Y axis. Line overlapping for positive and negative inclinations is related to magnitude of the acceleration that, for the Z axis, is only related to the inclination angle, rather than its orientation. For a null inclination (i.e., X, Y horizontal axes and vertical Z axis), the raw acceleration is null in the horizontal plane and corresponds to the magnitude of the gravity acceleration along the vertical axis.

It is interesting to note that, in the range of −10 t 45 °C, all of the lines of Figure 10 are approximately parallel each other but not horizontal. This suggest the existence of a thermal drifting and its apparent independence by the inclination or, in other words, by the magnitude of the acceleration. The average variation from the real value (drift) that, as indicated by the manufacturer, is the acceleration registered at 25 °C, is around 1300 LSB for the X axis and about 850 LSB for the Y axis. Additionally, it is possible to observe a decrease in the spacing of the lines for higher values of inclination connected to the known sensitivity deterioration of the accelerometer for high value of the acceleration [8]. 

It is also observable that the X and Y axes suffer from a measuring offset. It is highlighted by a shifting of the lines along the Y-axis of the graphs and can be evaluated in −1857 and −1823 LSB for the X axis during cooling and warming, respectively, and in −110 and −143 LSB for the Y axis during the same thermal cycles.

Conversely from X and Y axes, the Z axis exhibits a different and more complex behavior. It is characterized by the presence of multiple ripples [41] whose amplitude increase as the internal temperature approach that external and successively decrease. This occurs during both warming and cooling cycles. Despite their complexity, the curves are approximately horizontal. That’s why this behavior seems to be not indicative of a temperature-modulated drifting. We considered that this particular shape of the curves might be connected to the sensitivity of the sensor, not only to temperature variation, but also to the rate of variation over time. In our case, the high velocity of temperature variation within the chamber (2 °C/13 s, see Methods section) might be considered as the cause of this behavior. Additionally, similarly to the X and Y axes, the curve spacing variate as function of the inclination due to the sensitivity of the sensor described above.

### 4.2. Surfaces of Compensation and Associated Error

**Figure 12:**
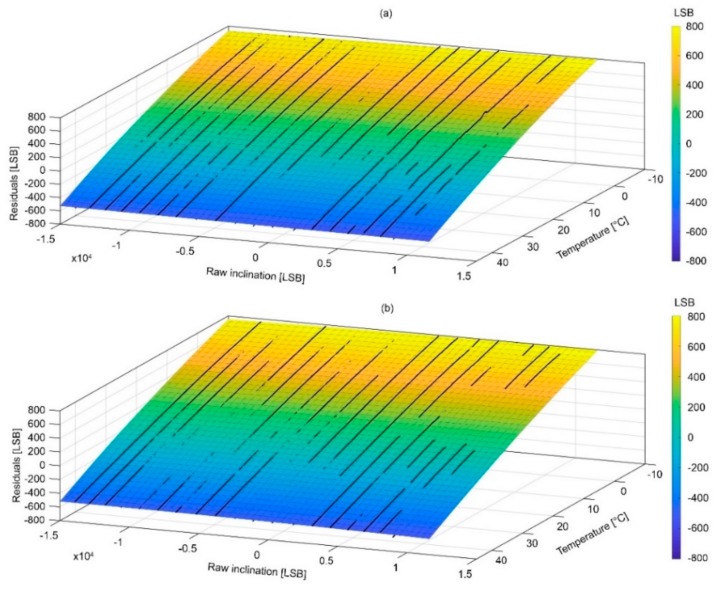
Graphs showing surfaces of compensations for the X axis during (**a**) warming and (**b**) cooling.

**Figure 13:**
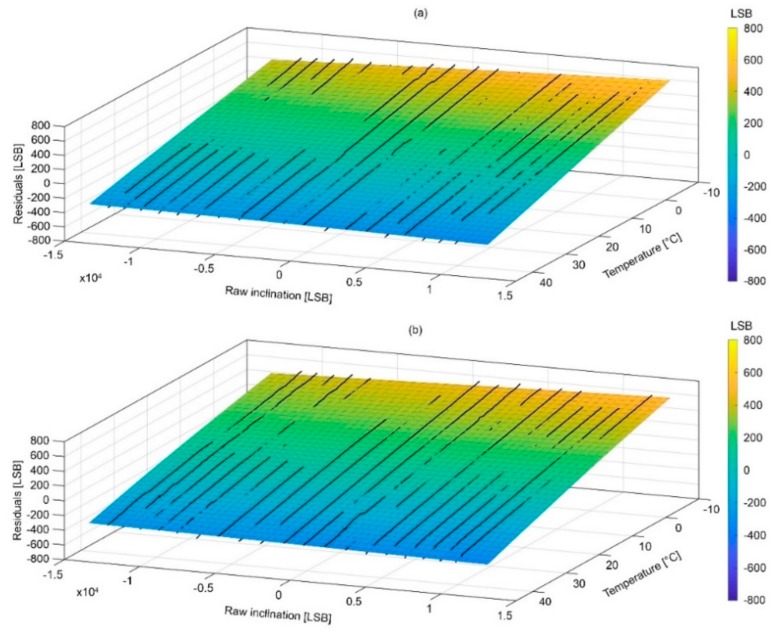
Graphs showing surfaces of compensations for the Y axis during (**a**) warming and (**b**) cooling.

**Figure 14:**
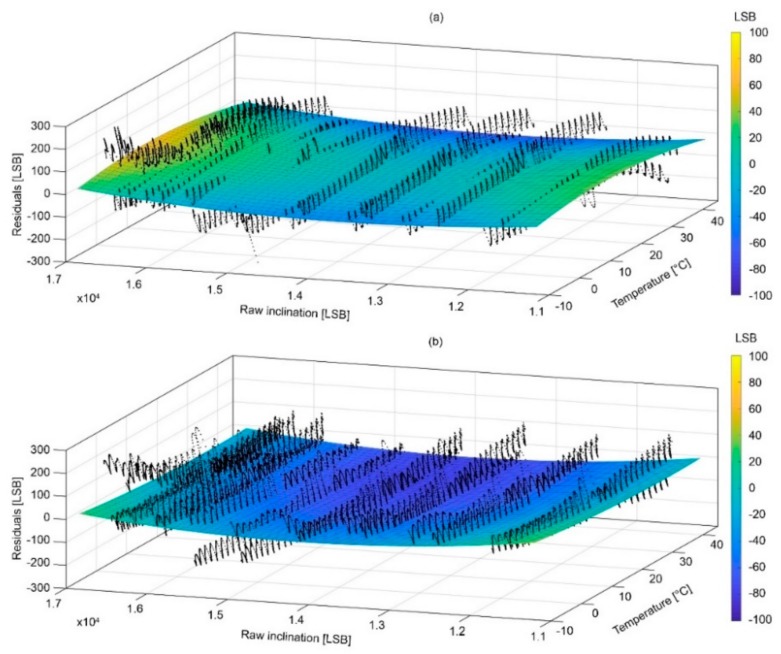
Graphs showing surfaces of compensations for the Z axis as function of the inclination of the X axis during (**a**) warming and (**b**) cooling.

**Figure 15:**
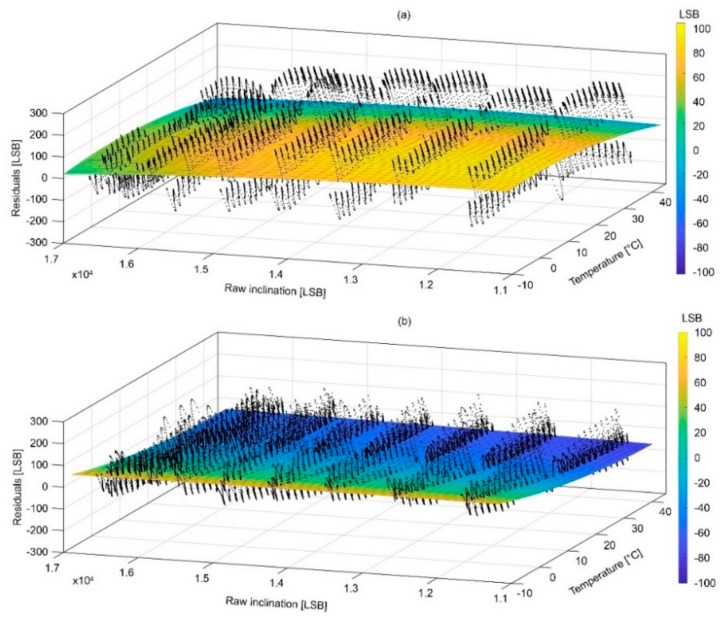
Graphs showing surfaces of compensations for the Z axis as function of the inclination of the Y axis during (**a**) warming and (**b**) cooling.

Figure 12 and Figure 13 show the compensation surfaces representing polynomial equations fitted on raw acceleration data measured along the X and Y axes, respectively, temperature and inclination. Figure 14 and Figure 15 show the same surface for the Z axis as function of inclination of the X and Y axes, respectively. Surfaces (a) refer to warming cycle and surfaces (b) refer to cooling cycles. General polynomial equation representing surfaces of compensation is reported below:

*f*(*x*,*y*) = *p*00 +10*x* + *p*01*y* + *p*20*x*^2^ + *p*11*xy* + *p*02*y*^2^ (3)

In Equation (3), x and y are the temperature and inclination. Surfaces parameterization, goodness of fit and RMS errors are reported in Table 2, Table 3, Table 4 and Table 5. It is important to notice how final RMS errors for the X and Y axes are consistently lower than those of the Z axis.

### 4.3. Compensation Application and Evaluation

**Figure 16:**
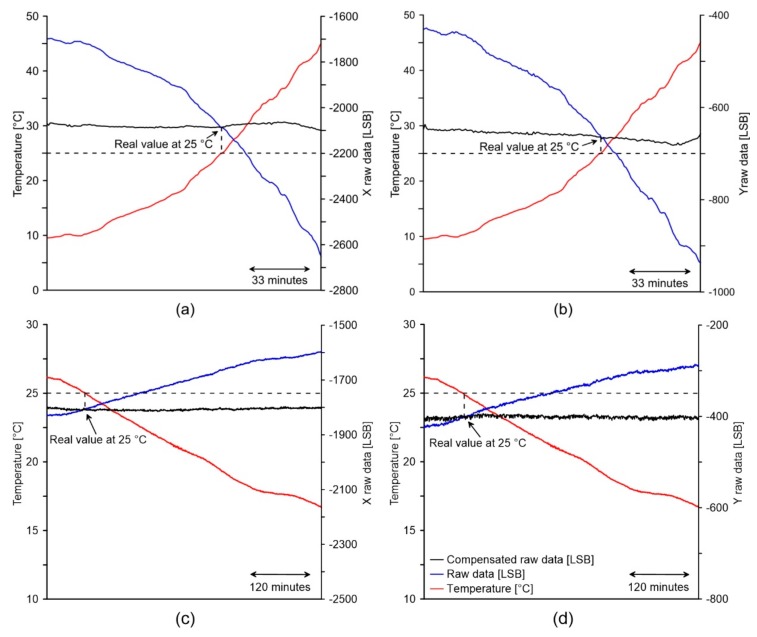
Graphs showing compensation testing during warming for (**a**) the X and (**b**) the Y axes and cooling for (**c**) the X and (**d**) the Y axes. Graphs legend is reported in (**d**).

The graphs of Figure 16 show time series of temperature, raw acceleration and compensated acceleration data for the (a, c) X and (b, d) Y axes, acquired during the field tests. Red and blue lines of graphs (a) and (b) indicate how temperature rises from 9 to 48 °C (red line) caused the thermal drifting of both the X and Y axes with a variation of the raw acceleration from −2653 to −1699 LSB and from −937 to −431 LSB, respectively. The RMS errors calculated considering the acceleration value at 25 °C and these data was estimated in 284 and 161 LSB for the X and Y axes, respectively. Similarly, the lines of graphs (c) and (d) indicate how the temperature fell from 26 to 16 °C (red line) caused the thermal drifting of both the X and Y axes with a variation of the raw acceleration from −1831 to −1596 LSB and from −424 to −286 LSB, respectively. In this second case, the RMS errors was estimated in 122 and 70 LSB for the X and Y axes, respectively.

The black lines of the graphs show the variation of the raw acceleration after the application of fitted polynomial equations for compensation purpose. Especially, in the case of rising temperature, thermal compensation of monitoring data was completed using polynomial equations parameterized as shown in Table 2 and Table 3 in the “warming” column. In the case of falling temperature, polynomial equations were parameterized as shown in Table 2 and Table 3 in the “cooling” column. After compensation the RMS errors related to the warming test decreased to 11 for X axis and 8 for Y axis, while the RMS errors related to the cooling test decreased to 4 for X axis and 4 for Y axis. Such a decrease indicates the performance of our model and how our procedure can improve precision of low cost accelerometers for tilt-based monitoring applications in field conditions. 

**Figure 17:**
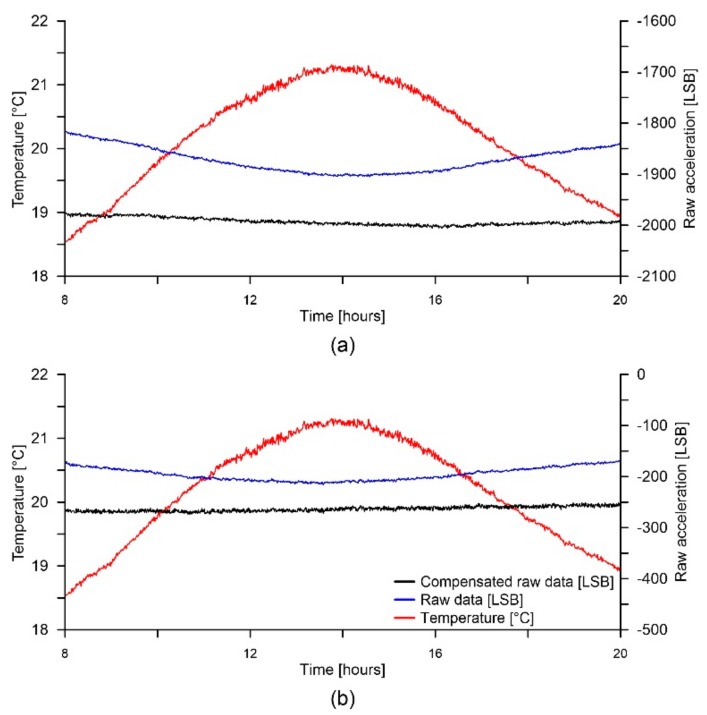
Graphs showing compensation behavior during an alternation between warming and cooling for (**a**) the X and (**b**) the Y axes. Graphs legend is reported in (**b**).

The graphs of Figure 17 show data acquired while alternating between a warming and a cooling cycle. The key problem of thermal compensation is to obtain a repeatable and stable thermal drift model able to operate in condition of thermal hysteresis, occurring when the warming cycle alternates with the cooling cycle. In this condition, although the thermal drifting shownd in Figure 17 is less evident in comparison with that of graphs of Figure 16 due to the smaller temperature variation, the figure seems to confirm the compensation performance of the strategy and suggest the stability of the model in condition of thermal hysteresis. 

Additionally, we expect the same behavior of the sensor during the alternation between a cooling and a warming cycle.

## 5. Conclusions

Low-cost MEMS accelerometric sensors have the potential to replace high precision electromechanical sensor in many tilt-based monitoring applications where very high resolution is not required. Despite their advantages, MEMS accelerometers have the disadvantage of being very sensitive to temperature variations, suffering from thermal drift that induces error in the acceleration measurements. To provide a simple compensation strategy the can be used in most monitoring applications, we have performed a thermal calibration analysis of onboard accelerometers of a low-cost MEMS IMU and used the raw data to construct low degree polynomial fits for thermal compensation. Each polynomial equation represents a compensation surface that relates the measurement error induced by thermal drift (i.e., acceleration residuals) to the temperature and inclination of the specific axis.

For thermal calibration analysis, we developed an Arduino^®^ controlled thermal chamber based on a Peltier element. The chamber, mounted on a tilting device, allowed the simulation of thermal behavior of onboard sensors at different inclinations. Thermal calibration was completed in the range −10 to +45 °C both during warming and cooling cycles. Following the manufacturer’s indications, we assumed that the acceleration measurement at 25 °C was not affected by measurement error or this error was negligible. On this basis, we used calibration data to calculate acceleration residuals, which were fitted, as well as temperature and inclination data, using Matlab™ to obtain compensation equations. For each axis, we obtained two equations (i.e., surfaces), a first applicable in warming condition (temperature rising) and a second for cooling conditions (temperature decrease). This in order to account for possible thermal hysteresis of the system. 

To validate our compensation strategy, we performed a field monitoring application and evaluated the compensation performance by calculating RMS errors before and after compensation. After compensation the RMS errors calculated for both the X and Y axes decreased of one order of magnitude and an average of 96%. This result of compensation of real monitoring data, shows the potential of using a simple set of equations to solve common drawbacks that hinder the applicability of low cost sensors for tilt-based monitoring applications. Additionally, our calibration underlined the particular behavior of the Z axis during temperature variations that we suppose to be related to its sensitivity to variation velocity. On this basis, we suggest that inclination computation should be based only on data from the X and Y axes.

## Figures and Tables

**Table 1:** Registers of the LSM9DS0 IMU and related settings.

Register	Setting
CTRL_REG1_XM	100 Hz accelerometer refresh rate
CTRL_REG2_XM	Set 50 Hz anti-aliasing filter
CTRL_REG5_XM	Enable temperature sensor

**Table 2:** Parameterization of polynomial equations for X axis compensation.

Warming		Cooling	
Coefficients (95% Conf. Bounds)		Coefficients (95% Conf. Bounds)	
p00	571.5 (571.5, 571.6)	p00	582.9 (582.7, 583.1)
p10	−20.23 (−20.24, −20.23)	p10	−21.29 (−21.3, −21.27)
p01	1.569 × 10^−3^ (1.56 × 10^−3^, 1.579 × 10^−3^)	p01	7.23 × 10^−4^ (7.535 × 10^−4^, 7.91 × 10^−4^)
p20	−0.1044 (−0.1045, −0.1042)	p20	−7.898 × 10^−2^ (−7.933 × 10^−2^, −7.863 × 10^−2^)
p11	−6.936 × 10^−5^ (−6.972 × 10^−5^, −6.9 × 10^−5^)	p11	−6.362 × 10^−5^ (−6.435 × 10^−5^, −6.29 × 10^−5^)
p02	6.327 × 10^−9^ (5.275 × 10^−9^, 7.38 × 10^−9^)	p02	−7.354 × 10^−8^ (−7.563 × 10^−8^, −7144 × 10^−8^)
**Goodness of fit**		**Goodness of fit**	
SSE	2.739 × 10^6^	SSE	1.043 × 10^7^
R^2^	0.9998	R^2^	0.9992
Adjusted R^2^	0.9998	Adjusted R^2^	0.9992
RMS error	6.256	RMS error	12.32

**Table 3:** Parameterization of polynomial equations for Y axis compensation.

Warming		Cooling	
Coefficients (95% Conf. Bounds)		Coefficients (95% Conf. Bounds)	
p00	354 (353.8, 354.2)	p00	338.1 (337.9, 338.2)
p10	−13.96 (−13.98, −13.95)	p10	−12.82 (−12.83, −12.81)
p01	5.747 × 10^−3^ (5.724 × 10^−3^, 5.77 × 10^−3^)	p01	4.439 × 10^−3^ (4.426 × 10^−3^, 4.453 × 10^−3^)
p20	−2.931 × 10^−3^(−3.369 × 10^−3^, −2.493 × 10^−3^)	p20	−3.299 × 10^−2^ (−3.325 × 10^−2^, −3.27 × 10^−2^)
p11	−1.915 × 10^−4^ (−1.925 × 10^−4^, −1.91 × 10^−4^)	p11	−1.579 × 10^−4^ (−1.584 × 10^−4^, −1.57 × 10^−4^)
p02	−5.363 × 10^−8^ (−5.633 × 10^−8^, −5.09 × 10^−8^)	p02	−1.455 × 10^−8^ (−1.613 × 10^−8^, −1.29 × 10^−8^)
**Goodness of fit**		**Goodness of fit**	
SSE	2.381 × 10^7^	SSE	7.744 × 10^6^
R^2^	0.9954	R^2^	0.9984
Adjusted R^2^	0.9954	Adjusted R^2^	0.9984
RMS error	17.37	RMS error	10.03

**Table 4:** Parameterization of polynomial equations for ZX axis compensation.

Warming		Cooling	
Coefficients (95% Conf. Bounds)		Coefficients (95% Conf. Bounds)	
p00	1166 (1131, 1201)	p00	1499 (1453, 1546)
p10	1.856 (1.659, 2.052)	p10	−1.729 (−1.994, −1.463)
p01	−0.1738 (−0.1788, −0.1688)	p01	−0.2198 (−0.2264, −0.2132)
p20	−0.06327 (−0.06462, −0.06192)	p20	0.02335 (0.02152, 0.02518)
p11	−1.532 × 10^−5^ (−2.831 × 10^−5^, −2.32 × 10^−6^)	p11	1.106 × 10^−5^ (−6.469 × 10^−6^, 2.858 × 10^−5^)
p02	6.387 × 10^−6^ (6.212 × 10^−6^, 6.562 × 10^−6^)	p02	7.801 × 10^−6^ (7.569 × 10^−6^, 8.033 × 10^−6^)
**Goodness of fit**		**Goodness of fit**	
SSE	1.675 × 10^8^	SSE	2.895 × 10^8^
R^2^	0.456	R^2^	0.03082
Adjusted R^2^	0.456	Adjusted R^2^	0.03074
RMS error	48.92	RMS error	64.9

**Table 5:** Parameterization of polynomial equations for ZY axis compensation.

Warming		Cooling	
Coefficients (95% Conf. Bounds)		Coefficients (95% Conf. Bounds)	
p00	−195.6 (−250.4, −140.7)	p00	−53.65 (−76.05, −31.26)
p10	−1.516 (−1.832, −1.201)	p10	−4.726 (−4.855, −4.597)
p01	0.04766 (0.0399, 0.05543)	p01	0.009953 (0.006769, 0.01314)
p20	−0.05595 (−0.05815, −0.05375)	p20	0.03852 (0.03763, 0.03941)
p11	1.354 × 10^−4^ (1.145 × 10^−4^, 1.563 × 10^−4^)	p11	8.257 × 10^−5^ (7.409 × 10^−5^, 9.104 × 10^−5^)
p02	−2.013 × 10^−6^ (−2.28 × 10^−6^, −1.74 × 10^−6^)	p02	−3.241 × 10^−7^ (−4.363 × 10^−7^, −2.12 × 10^−7^)
**Goodness of fit**		**Goodness of fit**	
SSE	6.002 × 10^8^	SSE	8.949 × 10^7^
R^2^	0.1251	R^2^	0.6632
Adjusted R^2^	0.125	Adjusted R^2^	0.6632
RMS error	87.21	RMS error	34.11

## Supplementary Materials

The following are available online at http://www.mdpi.com/1424-8220/18/8/2536/s1, Supplementary File A and B.
